# Sacituzumab tirumotecan (sac-TMT/MK-2870/SKB264): a novel antibody–drug conjugate in breast cancer

**DOI:** 10.3389/or.2026.1781533

**Published:** 2026-02-05

**Authors:** Amalia A. Sofianidi, Alkistis Papatheodoridi, Constantine Dimitrakakis, Spyridon Marinopoulos, Vasiliki Michalaki, Flora Zagouri

**Affiliations:** 1 Oncology Unit, Aretaieion University Hospital, National and Kapodistrian University of Athens School of Medicine, Athens, Greece; 2 Department of Clinical Therapeutics, Alexandra General Hospital of Athens, School of Medicine, National and Kapodistrian University of Athens, Athens, Greece; 3 First Department of Obstetrics and Gynecology, General Hospital of Athens Alexandra, School of Medicine, National and Kapodistrian University of Athens, Athens, Greece

**Keywords:** antibody drug conjugate (ADC), breast cancer, bystander effect, sacituzumab tirumotecan (MK-2870/SKB264), sac-TMT

## Abstract

The discovery of antibody-drug conjugates (ADCs) has revolutionized the therapeutic landscape of oncology patients, especially those suffering from breast cancer. Following the approval of the first ADC for solid tumors in breast cancer, numerous additional ADCs have also been launched in the therapeutic landscape of breast cancer and have become the new standard of care for all diverse subtypes. Sacituzumab tirumotecan (sac-TMT) (MK-2870/SKB264) is an innovative ADC targeting TROP2 and delivering a belotecan-derived topoisomerase I inhibitor payload. Several clinical trials of sac-TMT have demonstrated promising results including improved overall response and disease control rates as well as progression-free survival. The safety profile of sac-TMT seems easily manageable; adverse events include mainly grade 1/2 nausea and alopecia, while grade 3/4 neutropenia and leukopenia and grade 3/4 stomatitis have been also reported. However, no treatment-related deaths have been reported so far. Since sac-TMT offers an encouraging new option for diverse breast cancer populations with manageable toxicity profiles, this review aims to summarize the recent published data regarding its use in breast cancer.

## Highlights

- Antibody-drug conjugates (ADCs) are an advent in the management of oncology patients.
- Sacituzumab Tirumotecan (sac-TMT/MK-2870/SKB264) is a promising new ADC undergoing clinical trials.
- Favorable results have been published with the use of Sacituzumab Tirumotecan in diverse breast cancer subtypes.
- Patients with TNBC have derived the most promising clinical outcomes to date.

## 1 Introduction

The advent of Antibody-Drug Conjugates (ADCs) has shifted the therapeutic landscape of various solid and hematologic malignancies. ADCs currently constitute one of the most extensively investigated and expanding fields in medical oncology. Paul Ehrlich introduced the “magic bullet” concept in 1907, but only in the past decade ADCs have begun integrating into clinical practice (1). ADCs are often described as targeted therapies, but in essence, they function as targeted forms of chemotherapy. They comprise of a monoclonal antibody (mAb) directed against a tumor-associated antigen, a cytotoxic agent called payload, and a connecting linker. When the antibody recognizes the specific tumor antigen, it binds to the surface of the cancer cell and gets absorbed into the cell. Inside the cancer cell, the payload is released resulting in tumor cell death. During tumor cell death, certain ADCs can release their payload into the tumor microenvironment (TME), impacting nearby cells—a phenomenon called the bystander effect. Thus, they can be effective even if the tumor specific antigen is not expressed by every malignant cell (2).

The most prosperous application of ADCs amongst solid tumors has been in breast cancer; ADCs have become the new standard of care in the management of different breast cancer subtypes (3). Since the first FDA approval in 2013, four ADCs have been approved for breast cancer treatment until today. The first ADC that was approved for solid malignancies and more specifically for breast cancer, was Trastuzumab Emtansine (Kadcyla). Based on the results of the EMILIA study it was authorized for the treatment of human epidermal growth factor receptor 2 (HER2)-positive (HER2+) metastatic breast cancer in patients who had previously received trastuzumab and a taxane (4). KATHERINE was the trial that laid the groundwork for FDA approval of Kadcyla in the adjuvant setting for HER2+ early breast cancer patients with residual disease after neoadjuvant therapy (5). Trastuzumab Deruxtecan (T-DXd or Enhertu) is another anti-HER2 targeted ADC, approved in both HER2+ metastatic breast cancer (6) and HER2 low disease (7). In addition to HER2-directed ADCs, Trophoblast cell surface antigen 2 (TROP2)–targeted ADCs have also been approved for the management of breast cancer. Sacituzumab govitecan (Trodelvy) is a TROP2 targeted ADC currently approved in the management of metastatic triple-negative breast cancer (TNBC), alongside hormone receptor positive (HR+)/HER2 negative (HER2-) metastatic breast cancer based on the results of the ASCENT (8) and TROPiCS-02 studies, respectively (9). Recently, another TROP2 directed ADC, Datopotamab deruxtecan (Dato-Dxd), was granted FDA approval for the treatment of metastatic HR+/HER2- breast cancer (10). However, the need for further improvement in patient outcomes remains, particularly with respect to achieving greater efficacy while maintaining manageable off-target toxicity of ADCs. Accordingly, novel ADCs are being actively investigated in breast cancer; the most commonly diagnosed malignancy in women (11), with an observed trend toward a decreasing age at diagnosis (12).

When designing a novel ADC, choosing the most suitable target is of outmost importance. TROP2 is a type I cell surface glycoprotein, encoded by *tumor-associated calcium signal transducer 2* (*TACSTD2)* gene. Its expression remains absent or minimal in normal and healthy tissues, but it is upregulated across different tumor cells (13). This selected expression of TROP2 in malignant cells has made it a remarkable candidate when designing a new ADC. According to data from the Human Protein Atlas, TROP2 exhibits high expression levels in breast cancer tissue (14). Additionally, its expression levels are associated with poor prognosis; in TNBC particularly it was found to be positively correlated with higher TNM staging (13).

In breast cancer, novel ADCs targeting TROP2 have recently been developed. Sacituzumab tirumotecan (sac-TMT)—also known as MK-2870 or SKB264—is considered one of the most promising candidates, with satisfying results to date in breast cancer management. Sac-TMT consists of a humanized mAb targeting TROP2 protein, a novel linker, 2-methylsulfonyl pyrimidine, and a belotecan-derivative topoisomerase I inhibitor acting as the payload (15). The average Drug-to-antibody Ratio (DAR) is 7.4, which is close to the DAR achieved by standard cysteine conjugation (15). The key distinction of sac-TMT from other TROP2–targeting ADCs lies in its novel linker, which enhances stability in circulation and minimizes off-target toxicities (15). Sac-TMT specifically identifies TROP2 in the surface of cancer cells, becomes internalized and as a result, the payload is released intracellularly, generating DNA damage and eventually, cell death. Bystander effect is also evoked, leading neighboring cells to death (15). Sac-TMT is currently being investigated for several types of tumors in clinical trials, such as breast, endometrial, esophageal and non-small cell lung cancer (NSCLC).

The aim of this review is to highlight recent advances in the development and application of sac-TMT across different breast cancer subtypes.

## 2 Sac-TMT in breast cancer clinical trials

To date, two TROP2-targeting ADCs have been approved for use in breast cancer; Sacituzumab govitecan and Dato-dxd (8–10). The idea of further enhancing patient outcomes, while simultaneously ameliorating toxicity led to the designation of sac-TMT by Kelun-Biotech Pharmaceuticals in China. Subsequently, Kelun-Biotech granted Merck the exclusive rights to develop, manufacture and commercialize sac-TMT in domains outside of China.

The initial clinical trial investigating sac-TMT in clinical practice is a phase I/II study (MK-2870-001, NCT04152499), which recruited patients with unresectable or metastatic solid tumors that have proven refractory to standard treatment options. Currently, this study is active, but has completed accrual, enrolling 1,410 patients, including TNBC and HR+/HER2− breast cancer subtypes. The initial 2021 interim analysis included 18 patients across three dose levels (2, 4, and 6 mg/kg) and revealed that 2 out of 5 patients with TNBC had achieved partial response (PR) per RECIST 1.1. Among 17 patients assessed, the Overall Response Rate (ORR) was 35.3% (6/17), and the Disease Control Rate (DCR) was 70.6% (12/17). Treatment-related adverse events (TRAEs) were observed in all patients, mostly nausea grade 1 or 2 (72.2%) and alopecia (66.7%). Grade 3 or 4 TRAEs included neutropenia (27.8%), leukopenia (22.2%) and less frequently anemia (16.7%). Notably, no TRAEs leading to death were reported (16).

A cohort of the previous study included a subject of patients with HR+, HER2- or HER2 low metastatic breast cancer who received sac-TMT at a dose of 5 mg/kg every 2 weeks until progression disease (PD) or intolerable toxicity. Patients included had progressed on endocrine treatment and had received at least one line of chemotherapy in the metastatic setting. During the interim analysis in 2023, 41 patients were enrolled and had been followed up for a timeline of 8.2 months. 38 patients were suitable for tumor assessment. 14 of them achieved an ORR of 36.8%; PR was confirmed in 12. Median Duration of Response (DoR) was 7.4 months (range: 4.2–14.9+) and median progression-free survival (PFS) was 11.1 months (5.4–13.1). Notably, DCR was 89.5%. TRAEs aligned with previous published results; Grade 3 or 4 neutropenia (36.6%), leukopenia (22%), anemia (14.6%), platelet count decrease (9.8%) and gamma-glutamyl transferase (GGT) elevation (7.3%). The reported TRAEs led to a dose reduction for 17.1% of the patients; however, no TRAEs led to treatment withdrawal or death. Additionally, no neuropathy, incidents of pneumonitis or interstitial lung disease (ILD) were reported (17). Satisfying anticancer activity of sac-TMT has been observed in patients with previously treated HR+, HER2- or low metastatic breast cancer.

The most recent reported data from NCT04152499 phase I/II trial included 30 patients who were enrolled in the phase I cohort and received sac-TMT at different dose levels: 2 mg/kg (n = 4), 4 mg/kg (n = 7), 5 mg/kg (n = 7), 5.5 mg/kg (n = 5), and 6 mg/kg (n = 7). Five patients had dose-limiting toxicities (DLTs): grade 3 stomatitis at 4, 5.5, and 6 mg/kg; grade 3 rash at 5 mg/kg; and grade 3 urticaria at 6 mg/kg. Maximum Tolerating Dose (MTD) was 5.5 mg/kg and Recommended Doses for Expansion (RDEs) were 4 and 5 mg/kg. In the phase II dose expansion cohort, ORR was 34.8% (95% CI: 16.4%–57.3%) in the 4 mg/kg group (n = 23) and 38.9% (95% CI: 23.1%–56.5%) in the 5 mg/kg group (n = 36) for TNBC. Additionally, ORR was 31.7% (95% CI: 18.1%–48.1%) amongst the 41 patients with HR+/HER2- breast cancer who received sac-TMT (18). Most phase III trials of sac-TMT have adopted a dose of 4 mg/kg to be tested across different malignancies.

It is worth mentioning that sac-TMT is approved in China by National Medical Products Administration (NMPA) as a treatment option for patients with locally advanced or metastatic TNBC who have already received at least two prior chemotherapy regimens, including one at an advanced stage. A phase II study of sac-TMT conducted in China (NCT05445908, OptiTROP-Breast05) assessed this novel ADC with or without a programmed death-ligand-1 (PD-L1) inhibitor as first line option for patients with either locally advanced/metastatic TNBC or HR+/HER2− breast cancer. In this trial, the dose regimen of 5 mg/kg every 2 weeks was selected. In November 2024, with a median follow-up period of 18.6 months, 41 patients were registered. An ORR of 70.7% was established, while the DCR reported was 92.9%. Median DoR was 12.2 months and median PFS 13.4 months. Alike encouraging results were noted in the subgroup of patients with a Combined Positive Score (CPS) of less than 10, defined as PD-L1 negative disease with IHC 22C3 pharmDx assay. Most common Grade 3 or 4 TRAEs were neutropenia (46.3%), leukopenia (34.1%), anemia (12.2%) and stomatitis (9.8%). Again, no TRAEs led to death (19). The interim results of this phase II study presented during the American Society of Clinical Oncology (ASCO) 2025 meeting are intriguing; Sac-TMT demonstrated a favorable antitumor activity in patients with advanced or metastatic TNBC and negative PD-L1 expression. It should be noted that this trial paves the way for new and effective treatment options in TNBC patients; indeed, PD-L1 negative TNBC are less responsive to neoadjuvant chemotherapy and are linked to worse survival rates compared to patients with PD-L1 positive tumors (20).

Phase III clinical trials are currently underway, investigating the antitumor potential of sac-TMT in breast cancer patients. The research focus has shifted towards patients with TNBC based on the encouraging results of phase II trials in this breast cancer subpopulation. NCT05347134 is a phase III study comparing sac-TMT monotherapy to physician’s choice of chemotherapy in patients with heavily pretreated advanced TNBC. Based on the data extracted from the first interim analysis in 2023, a hazard ratio (HR) of 0.31 was determined (95% CI: 0.22–0.45, p < 0.00001). The median PFS was 5.7 months (95% CI: 4.3–7.2) with the new ADC and 2.3 months (95% CI: 1.6–2.7) with standard chemotherapy; PFS at 6 months was 43.4% amongst patients who were randomized to sac-TMT vs. 11.1% in the control arm, results favoring sac-TMT implementation in clinical practice. With the aim of facilitating biomarker implementation in clinical practice, TROP2 expression was also measured using immunohistochemistry (IHC); patients with high TROP2 expression (TROP2 H-score > 200) achieved a median PFS of 5.8 months with sac-TMT versus 1.9 months with chemotherapy (HR 0.28; 95% CI: 0.17–0.48), results that do not differ with the overall population. With a median follow-up of 10.4 months, OS was statistically significant in favor of sac-TMT (HR 0.53; 95% CI: 0.36–0.78; p = 0.0005). ORR was determined at 43.8% with sac-TMT and 12.8% with traditional chemotherapy regimens. Grade 3 or 4 TRAEs were reported in alignment with the previously published results; neutropenia, anemia and leukopenia were most frequently reported. Nevertheless, the rate of leukopenia and neutropenia was lower in the sac-TMT arm compared to the chemotherapy arm (25.4% vs. 36.4% and 32.3% vs. 47%, respectively) (21). Newest data was published in April 2025; the median PFS was determined at 6.7 months (95% CI: 5.5–8) at the group that received sac-TMT and 2.5 months (95% CI: 1.7–2.7) at the chemotherapy group (HR 0.32, 95% CI: 0.24–0.44, p < 0.00001), while the ORR was 45.4% with sac-TMT and 12% with chemotherapy (21, 22). Given the promising results of this novel ADC in TNBC, a phase III study (MK-2870-011/TroFuse-011, NCT06841354) evaluating sac-TMT as first line treatment modality in these patients has been launched.

Several other trials are ongoing, evaluating sac-TMT in breast cancer patients in different treatment settings. Table 1 presents an overview of the ongoing clinical trials evaluating sac-TMT in breast cancer patients, alongside the preliminary results published to date, if available.

**TABLE 1:** Summary of the ongoing clinical trials evaluating sac-TMT in breast cancer patients.

Clinical trial ID	Disease	Phase	Drugs	Pts	Status	Preliminary results	TRAEs
NCT04152499 (MK-2870-001)	Unresectable/met solid tumors	I/II	sac-TMT	1,410	Active, completed accrual	First interim analysis (2021) TNBC: 2/5 PR HER2+BC: 1/1 PR ORR: 35.3% (6/17 pts) DCR: 70.6% (12/17 pts)	Grade 3/4: Neutropenia (27.8%), leukopenia (22.2%), anemia (16.7%) Grade 1/2: Nausea (72.2%), alopecia (66.7%) (16)
	Cohort I: HR+, HER2- or low met BC	​	sac-TMT 5 mk/kg q2w	​	​	Interim analysis (2023) ORR: 36.8% (14/38 pts) PR: confirmed in 12 pts, unconfirmed in 2 pts Median DoR: 7.4 months (range: 4.2–14.9+) Median PFS: 11.1 mo (95% CI 5.4–13.1) DCR: 89.5%	Grade 3/4: Neutropenia (36.6%), leukopenia (22%), anemia (14.6%), thrombocytopenia (9.8%), GGT elevation (7.3%) (17)
	Cohort II: TNBC	​	​	​	​	June 2025 data ORR (TNBC): 34.8% (95% CI: 16.4%–57.3%) (4 mg/kg) group (n = 23) and 38.9% (95% CI: 23.1%–56.5%) (5 mg/kg) group (n = 36) ORR: 31.7% (95% CI: 18.1%–48.1%) for HR+/HER2- BC (n = 41)	DLTs: grade 3 stomatitis at 4, 5.5, and 6 mg/kg, grade 3 rash at 5 mg/kg, grade 3 urticaria at 6 mg/kg (18)
NCT05445908 (OptiTROP-Breast05)	Locally adv/met TNBC/HR+/HER2- BC	II	sac-TMT +/− PD-L1 inhibitor	175 (est)	Unknown status	Data cut off point (November 2024) ORR: 70.7% (29/41 pts, with 3 unconfirmed PRs) DCR: 92.9% Median DoR: 12.2 months Median PFS: 13.4 months	Grade 3/4: neutropenia (46.3%), leukopenia (34.1%), anemia (12.2%), stomatitis (9.8%) (19)
NCT07054242	Treatment naïve stage II/III TNBC	II	sac-TMT + pembro	52 (est)	Not yet recruiting	n/a	n/a
NCT05347134	Adv TNBC	III	sac-TMT vs. TPC	254 (est)	Active, not recruiting	First interim analysis (2023) Hazard ratio: 0.31 (95% CI: 0.22–0.45, p < 0.00001) Median PFS: sac-TMT: 5.7 months (95% CI: 4.3–7.2) vs. chemo: 2.3 months (95% CI: 1.6–2.7) Median OS: sac-TMT not reached [95% CI: 11.2- NE] vs. chemo: 9.4 months (95% CI: 8.5–11.7), HR 0.53; 95% CI: 0.36–0.78; p = 0.0005 ORR: 43.8% with sac-TMT vs. 12.8% with chemotherapy	Grade 3/4 (Sac-TMT vs. chemotherapy): neutropenia (32.3% vs. 47.0%), anemia (27.7% vs. 6.1%), leukocytopenia (25.4% vs. 36.4%) (21)
						Updated data (04/2025) Median PFS: sac-TMT: 6.7 months (95% CI: 5.5–8) vs. chemo: 2.5 months (95% CI: 1.7–2.7) (HR 0.32, 95% CI: 0.24–0.44, p < 0.00001) Median OS: sac-TMT: not reached (95% CI: 11.2-NE) vs. chemo 9.4 months (95% CI: 8.5–11.7) (HR 0.53; 95% CI: 0.36–0.78; p = 0.0005) ORR: MK-2870: 45.4% vs. chemo: 12% Median DoR: sac-TMT: 7.1 mo (95% CI: 5.6–NE) vs. chemo: 3 months (95% CI: 2.5–NE)	Grade 3/4: neutropenia (34.6% with sac-TMT and 47.0% with chemotherapy), anemia (29.2% and 6.1%), leukocytopenia (27.7% and 36.4%) (21, 22)
NCT06312176 (TroFuse-010)	Adv/met HR+/HER2- BC	III	sac-TMT +/− pembrolizumab vs. TPC	1,200 (est)	Recruiting	n/a	n/a
NCT06393374 (MK-2870–012)	Early TNBC pts with residual disease after NACT	III	sac-TMT + pembrolizumab vs. TPC	1,530 (est)	Recruiting	n/a	n/a
NCT06966700	Early-stage HR-low+/HER2- BC or TNBC	III	NACT sac-TMT + pembro followed by carboplatin/Paclitaxel/Pembro vs. chemo + pembro	2,400 (est)	Recruiting	n/a	n/a
NCT06841354 (TroFuse-011)	Adv/Met TNBC with CPS<10	III	sac-TMT +/− pembro vs. TPC	1,000 (estimated)	Recruiting	n/a	n/a
NCT07054242	Stage II/III TNBC	II	sac-TMT + pembro	52 (est)	Not yet recruiting	n/a	n/a

Pts: patients, met: metastatic, BC, breast cancer, pos: positive, neg: negative, est: estimated, adv: advanced, CI, confidence interval; CPS, combined positive score; DCR, disease control rate; DLTs, dose-limiting toxicities, DoR, duration of response, GGT, gamma-glutamyl transferase; HR, hormone receptor; ORR, objective response rate; pCR, pathologic complete response; PD-L1, programmed death-ligand 1; PFS, progression-free survival, pts, patients, PR, partial response; sac-TMT, sacituzumab tirumotecan; TNBC, triple-negative breast cancer; TPC, treatment of physician’s choice, TRAEs, treatment-related adverse events, vs., versus, pembro: pembrolizumab, chemo: chemotherapy, mo: months.

## 3 Discussion-future perspectives

Currently, ADCs represent a new era of targeted oncology. They represent clinically mature therapeutic options with substantial survival benefits in different solid tumors. Indeed, other targeted approaches have been designed, such as antibody guided nanoparticulate drug delivery systems. Although these systems offer greater flexibility in payload type and loading capacity, enabling delivery of different molecules, they face challenges; *in vivo* stability, bioavailability, and obstacles in delivery due to biological barriers are areas that require further development (23). Nevertheless, ADCs benefit from more predictable pharmacokinetics and established clinical development pathways, and this is the reason why the scientific community has invested so much in them.

With breast cancer being the leading malignancy amongst females, there is no doubt that ADCs have already provided meaningful clinical benefit and will likely continue to do so. For example, advanced TNBC has poor survival outcomes. The current standard treatment for patients with advanced TNBC includes chemotherapy alongside immune checkpoint inhibitors if PD-L1 positivity is verified (24). The latest data presented at ASCO 2025 supports the use of the anti-TROP2 targeted ADC Sacituzumab govitecan in combination with pembrolizumab as the new standard of care for advanced TNBC patients. With a median PFS of 11.2 months (95% CI: 9.3–16.7) in the ADC plus immunotherapy arm versus 7.8 (95% CI: 7.3–9.3) months in the chemotherapy plus immunotherapy arm, Sacituzumab govitecan will possibly be integrated into the first line setting of advanced TNBC management. Although OS data were early, a beneficial increase in OS rates was also detected (25). This hopeful announcement paves the way for the use of other TROP2 targeted ADCs in the management of breast cancer patients, with the hope of even enhanced patient outcomes.

In addition to the well-described Sacituzumab govitecan, newly described TROP2-targeting ADCs, such as Dato-Dxd and sac-TMT have been launched, challenging Trodelvy’s dominance. In the first-line setting for metastatic TNBC, Dato-Dxd showed PFS outcomes comparable to those reported for Sacituzumab govitecan, as observed in the TROPION-Breast02 and ASCENT-03 trials, respectively (26, 27). However, the safety profile of Dato-Dxd is more appealing; fewer TRAEs grade 3 or higher and no treatment-related deaths were reported. Hematologic toxicity also appears to be milder with Dato-Dxd (26). Comparing sac-TMT to Dato-Dxd is challenging, due to the immaturity of the published results of Sac-TMT. Their key differences lie in their formulation; Dato-dxd has a DAR of 4.0, while Sac-TMT has a DAR of 7.1, indicating that Sac-TMT is more potent. On the other hand, Dato-Dxd has a novel exatecan derivative as a payload, which is 10 times more potent than sac-TMT’s payload SN-38 in inhibiting topoisomerase I (28, 29). Phase III data is expected from trials with sac-TMT to directly compare it with Dato-Dxd. Enhancing patient outcomes, while minimizing toxicity is the pilar of oncology. Notably, sac-TMT has demonstrated a favorable safety profile across clinical trials. Hematologic toxicity is the most frequently TRAE detected at a grade ≥ 3, although manageable with the supportive use of growth factors. A high alopecia rate has also been observed with this ADC, consistent with findings reported for other ADCs (30). Alopecia has few physically harmful effects alopecia; however, it is a well-known side effect of chemotherapy as well that often requires sensitive management and support. Oral mucositis (stomatitis), a notable on-target, off-tumor toxicity of ADCs, is the main distinctive toxicity observed with sac-TMT. This unique TRAE can impact patient quality of life, impede nutritional intake, heighten infection risk, and result in dose modifications or ultimately drug discontinuation. Nevertheless, it should be noted that preventative measures are implemented with most ADCs, such as prophylactic cryotherapy, namely, ice chips or ice water held in the mouth during the infusion, and the use of steroid-containing mouthwash at home (31).

Significantly, most ADC toxicities are impelled by the payload’s effects on healthy tissues. For example, a major adverse event of Sacituzumab govitecan is diarrhea, which can easily be explained by the fact that its payload is the active metabolite of irinotecan (32). Gastrointestinal toxicity in terms of diarrhea is frequently reported with Sacituzumab govitecan; however, this TRAE which can be severe has not been observed with sac-TMT. Belotecan, which is the payload of sac-TMT, is known to cause myelosuppression, which explains the hematologic toxicity observed with this new ADC. Grade 3 or 4 non-hematologic adverse reactions of belotecan include dyspnea and anorexia, although not frequently observed (33). What remains to be elucidated is whether Sac-TMT is associated with ILD or pneumonitis like other ADCs and whether these events are also manageable. There may be a potential association between belotecan-associated dyspnea and ADC-induced pneumonitis, if observed in the future. To date, no such events have been reported (34).

An intriguing avenue for future research is the potential use of TROP2 expression as a stratification factor for administering TROP2-targeted ADCs. However, to date efficacy of Sacituzumab govitecan has been observed regardless of high TROP2 expression levels (35). The first published results from the phase III study (NCT05347134) of sac-TMT in patients with advanced TNBC confirm this observation; patients with high tissue TROP2 expression did not achieve a longer PFS comparing to patients with low tissue TROP2 expression (21). Nevertheless, TROP2 expression in breast cancer tissue may serve as a valuable marker for investigating resistance mechanisms or as a prognostic marker either for disease recurrence or for survival.

## 4 Conclusion

Ultimately, Sac-TMT is an emerging ADC, targeting the glycoprotein TROP2, which is highly expressed in cancer tissues. It has already shown clinical efficacy in the management of previously treated advanced Epidermal Growth Factor Receptor (EGFR)-mutated NSCLC (34) and is currently being investigated in several other malignancies. Promising efficacy results were observed amongst breast cancer patients, particularly those with TNBC. More data is expected, as several clinical trials are still ongoing. Indeed, ongoing research and clinical advances continue to refine the landscape of ADC therapy, fostering optimism for more effective and individualized treatment options in advanced breast cancer. The mechanisms of resistance for current ADCs and their optimal sequencing remain to be determined.
